# Early access to a cardio-oncology clinic in an Australian context: a qualitative exploration of patient experiences

**DOI:** 10.1186/s40959-022-00140-3

**Published:** 2022-08-09

**Authors:** Jennifer White, Julie Byles, Trent Williams, Rossana Untaru, Doan T. M. Ngo, Aaron L. Sverdlov

**Affiliations:** 1 Centre for Women’s Health Research, College of Health Medicine and Wellbeing, University of Newcastle, Callaghan Campus, University Drive, Callaghan, NSW 2308 Australia; 2 Hunter Medical Research Institute, Level 4 West Wing, Lot 1, Kookaburra Cct, New Lambton Heights, Newcastle, NSW 2305 Australia; 3 Hunter New England Local Health District, Lookout Rd, New Lambton, NSW 2305 Australia; 4 Newcastle Centre of Excellence in Cardio-Oncology, Calvary Mater Newcastle, Hunter Medical Research Institute, Hunter New England Local Health District and University of Newcastle, Newcastle, NSW Australia; 5 School of Biomedical Sciences and Pharmacy, College of Health Medicine and Wellbeing, University of Newcastle, Callaghan Campus, University Drive, Callaghan, NSW 2308 Australia; 6 School of Nursing and Midwifery, College of Health Medicine and Wellbeing, University of Newcastle, Callaghan Campus, University Drive, Callaghan, NSW 2308 Australia; 7 School of Medicine and Public Health, College of Health Medicine and Wellbeing, University of Newcastle, Callaghan Campus, University Drive, Callaghan, NSW 2308 Australia

**Keywords:** Qualitative, Cardio-oncology, Patient experience

## Abstract

**Background:**

Dedicated cardio-oncology services are emerging rapidly around the world in order to provide cardiovascular care (CV) for cancer patients. The perspectives of patients regarding their experience of cardiac surveillance during their cancer journey has not been qualitatively evaluated.

**Methods:**

An interpretative qualitative study. Fifteen, in-depth qualitative interviews were conducted with a diverse range of community dwelling patients who attended a newly established cardio-oncology clinic in a large regional city in Australia. Data were analysed using an inductive thematic approach.

**Results:**

Key themes were identified: (1) Access to a cardio-oncology clinic promotes information and understanding, (2) The experience of early CV intervention, (3) Factors promoting integrated care, (4) Balancing cancer treatment and CV symptoms and (5) Managing past and emerging CV risk factors.

**Conclusion:**

As cardio oncology clinics continue to emerge, this study confirms the benefit of early access to a cardiologist for management of existing or emerging CV risk factors and diseases in the context of cancer treatment. Participants valued the opportunity for regular monitoring and management of CV issues that enabled them to continue cancer treatment. However, we identified gaps in education and support towards making positive lifestyle changes that reduce the risk of CV diseases in cancer patients.

## Introduction

Historically, health care for people with cancer has focused on specific anti-cancer treatments aimed at improving cancer-specific outcomes. As a result, co-morbid health conditions have frequently taken secondary consideration. The occurrence of cardiotoxicity, a complication of many anti-cancer agents (both conventional chemotherapies and newer biological therapies), has been well-documented with current figures suggesting it occurs in up to one quarter of cancer patients [1–3]. Treatment-induced cardiotoxicity is defined as the direct effects of cancer treatment on cardiovascular (CV) function and structure [4, 5]. Many patients may survive cancer only to develop cardiovascular disease (CVD) which sometimes has a higher mortality rate than cancer itself [1, 4]. In response, cardio-oncology, as a dedicated specialty, has emerged with the focus on detection, monitoring, and treatment of CVD occurring in cancer patients.

Growing international support for cardio-oncology services [4, 6] has led to the development of guidelines to facilitate the delivery of a multidisciplinary cardio-oncology care that aims to help cancer patients remain on and complete their cancer treatment without interruption due to existing or emerging CVD. Such innovative health programs are important given the increasing challenge of navigating treatment across multiple comorbidities, including cancer, especially if given treatment recommendations are conflicting or lead to interactions [7].

Increasing rates of cancer survivorship due to improved cancer detection and treatments reinforce the imperative of ensuring that patients are educated towards making lifestyle choices that prevent CVD in the long term [8, 9]. Well established goals of reducing risk for both cancer and CVD include combatting smoking, sedentary lifestyle, alcohol consumption, poor diet and obesity [10]. Specifically, prevention is critical to reduce CVD risk after a cancer diagnosis [11].

In an Australian context, a study by Clark et al. (2019) explored the experience of cardiotoxicity at 3 large medical centres in 2 Australian states [12]. A review of medical records identified 50 patients with a confirmed diagnosis of cardiomyopathy from cardiotoxicity between 2004 and 2015. In that study cardiotoxicity was defined as left ventricular ejection fraction (LVEF) reduction of ≥15% from baseline despite normal function, or an LVEF decline to < 50%, or an LVEF decline considered clinically significant by the cardiologist [12]. During the study period, 39 (78%) of patients commencing cancer therapy had a least one CVD risk factor including hypertension, current smoking, high body mass index, hypercholesterinemia, diabetes and age over 65 years, with 24% having four or more modifiable CVD risk factors [12]. This profile is consistent with current levels of CVD risk factors in the Australian population [13]. Supporting qualitative interviews with patients identified gaps in service delivery whereby participants were not informed of the potential for cardiotoxicity prior to treatment, were not provided with CVD education and experienced a lack collaboration between their treating oncologists and cardiologists [12].

In line with growing evidence towards the need for dedicated cardio-oncology services, and the development of strategies to improve guideline-directed cardioprotective therapies in cancer patients and survivors, a comprehensive cardio-oncology service commenced at the Calvary Mater Hospital, a Level 6 Cancer hospital in Newcastle NSW, in 2018. The program focusses on early detection and risk stratification of cardiotoxicity, as well as management of pre-existing or de novo CV risk factors and CVD. In brief, the cardio-oncology service consists of weekly outpatient clinics, co-located with oncology outpatient clinics, conducted in parallel by the Cardiologist (ALS; experienced in cardio-oncology), Cardiology Fellow and cardio-oncology nurse (TW). Cardiologist and Fellow clinics see patients referred for a number of indications, including high risk patients for cardiovascular management and optimisation, suspected and established cardiovascular disease. Nurse-led cardio-oncology clinic focusses on cardiovascular risk stratification, management, education as well as lifestyle, pharmacological and non-pharmacological interventions. There are cross-referral pathways between the 3 clinics. There are also established electronic referral pathways for patients referred from cancer services with appointments allocated according to clinical need. Urgent referrals are seen within 1 week, and initial clinical advice is provided immediately over the phone when required. More urgent reviews (within 24–36 hours are also available depending on clinical need). Inpatient consultation service is also provided with patients seen on the day of referral.

Sustained implementation of innovative practice change is challenging and feedback from clinicians and patients regarding this is important [14]. To our knowledge there are no qualitative evaluations of the implementation of cardio-oncology services exploring patient experiences. Qualitative techniques provide an opportunity to explore experiences inductively in natural contexts and provide an opportunity for study participants to give detailed accounts of their experiences and present their own perspectives and interpretation of these experiences [15], which cannot be captured using quantitative methods [16]. The research question underpinning this study was: what are patients’ perceptions toward acceptability and feasibility of a Cardio-oncology services and its impact on integrated care?

## Methods

This qualitative study employed the use of semi-structured interviews and was informed by the Consolidated criteria for reporting qualitative research (COREQ) checklist [17]. For pragmatic reasons we used purposive sampling to identify participants of varying ages at differing stages of their cancer experiences to capture a diverse range of perspectives. Patients were eligible for inclusion if they were receiving services at our cardio oncology clinic and were not receiving palliative care. Specifically, we aimed to capture the experience of newly referred patients as well as patients who had received a minimum of three follow-up appointments and patients who have received seven or more follow-up appointments. Potential participants were identified by and invited to participate by their treating cardio-oncology nurse and/or cardio-oncologist. During their appointment participants were provided with detailed study information and had the opportunity to ask questions about the research. All invited participants agreed to participate. All participants provided written informed consent by return of the consent form to the Principal Investigator via email or post. Recruitment occurred between March and July 2021. Approval for this project was obtained from the Hunter New England Health Human Research Ethics Committee (Ref: **2020/ETH02363)**.

### Data collection

Semi-structured interviews with participants were conducted by a skilled qualitative interviewer (JW) at a mutually convenient time and location. The majority of participants opted to be interviewed in their own home, and one opted to be interviewed at a local café. Interviews were guided by an interview schedule (see Table 1) [18]. The interviewer was not associated with the implementation of the cardio-oncology service; this assisted in reduction of bias and facilitated open discussion that allowed participants to express their opinions. Interviews began by asking participants to share their experience of cancer and CVD and the challenges undergoing treatment for two health conditions. Subsequent questions explored perceptions of access to the cardio-oncology clinic including benefits, barriers and opportunities. Participants had the option of withdrawing from the study, ceasing or rescheduling the interview if they became distressed however this was not required. Identified themes informed continuing data collection and sampling continued until thematic saturation (two co-coders agreeing that no new themes were emerging) was achieved.

**Table 1:** Interview guide

Question	Prompt
Can you tell me a bit about your health condition and why you were referred to the cardio-oncology clinic?	What was the reason for your referral to the cardio-oncology clinic? Did you understand why you were being referred?
How did you respond to your doctor’s recommendation that your attend the cardio-oncology clinic?	How do you feel about this? Expand E.g. worried, anxious, confused? Were you overwhelmed at the thought of seeing another specialist? Expand
What was it like to realise you had more than one disease that needed medical attention?	Expand? What did you do? Emotions Decision making Prioritising Doing what was asked by 2 different doctors
What has been your experience of the cardio-oncology clinic?	Did you understand what was happening? Did you understand the connection with your cancer? How did that make you feel? Was it hard to follow the advice of 2 doctors? What would suggest to improve the service?
Do you have any other needs that you feel are not being addressed because of your cancer or heart disease?	How did treatment affect your life eg education, family, work, etc.?” What would have helped?
If you could change one thing about the healthcare services you received, what would it be?	
Do you have any further comments	

The interview duration ranged from 30 to 60 minutes. The interviews were audio-recorded, transcribed verbatim, and checked for transcription errors. Field notes were made immediately after the interviews to record observations, capture initial ideas on the topics, and reflect on the methodology (e.g., interview guide refinement). Hard copy data (i.e field notes) were stored in a locked filing cabinet and electronic files were password protected. Only the researchers coding the data had access to the field notes and transcripts.

### Data analysis

Two authors (JW, JB), one an occupational therapist and the other a gerontologist, independently coded data using an inductive thematic approach [19]. Inductive thematic analysis consists of six steps: familiarizing with the data, generating initial codes, searching for themes, reviewing themes, defining and naming themes, and writing [20]. At the level of initial coding both authors read the transcripts multiple times and made notes. Transcripts were then coded line-by-line, describing and interpreting emerging categories and searching for differences and similarities. Following team discussion, the primary author developed a single codebook and each code was issued with a four-letter label or code to facilitate data retrieval between the transcripts (for example feelings of confidence was labelled ‘CONF). The next step involved examining the relationship between codes in the context of the research question in order to form themes. Consistency of findings was upheld through discussion of interpretations between researchers to confirm codes and categories. Any differences in researcher perspective were resolved by negotiation and, if necessary regrouped and recoded until consensus were reached. New codes were then fed back into the analysis to cross-check codes and themes and develop an overall interpretation of the data [21]. Trustworthiness of our data was upheld using several strategies, including immersion in data; reflexive analysis, and peer debriefing [22, 23]. Coders captured exemplar quotes supporting each theme.

## Results

Participant demographics are outlined in Table 2. Fifteen participants (10 female, five male) participated in this study (age range: 38–74 years). Participants experienced between one and four CVD risk factors, and all were receiving cancer treatment.

**Table 2:** Patient characteristics

Patient identification number	Gender	Country of birth	Age	Highest level of education	Cancer Diagnosis	CVD Diagnosis	Number clinic visits
1	M	Australia	65	Lower secondary	Metastatic lung	Chronic AF HT Dyslipidemia	5
2	M	Australia	73	Lower secondary	Metastatic Melanoma	MVR & Atrial septal defect repair 2019 Heart Failure	9
3	F	Australia	61	Bachelor degree	Breast	HT Morbid obesity	10
4	F	Australia	47	High School Certificate	Breast	LVEF drop	6
5	M	Australia	57	Certificate	Melanoma metastatic	ACS HT LVH due to HT	4
6	F	Australia	49	Certificate	Metastatic breast	HT Left ventricular systolic dysfunction	2
7	F	Australia	64	Upper secondary	Sigmoid Metastatic breast	Minor CAD	2
8	M	United Kingdom	62	PhD	AL Amyloidosis	LVH	4
9	F	Australia	64	Bachelor degree	Breast	PHT PAF	5
10	F	New Zealand	43	Upper secondary	Breast	Ex smoker Overweight PCOS	2
11	F	Australia	38	Bachelor degree	Breast	Ex-smoker	2
12	F	Australia	63	Lower secondary	Follicular NHL	HFrEF	8
13	M	Australia	74	Bachelor degree	Metastatic clear cell, renal cell	MVR DCM AF	13
14	F	Australia	59	Certificate	Breast	HT Dyslipidemia NSTEMI	7
15	F	Australia	62	Yr 10	Breast	Dyslipidemia Osteoarthritis	6

*AF* Atrial Fibrillation, *CAD Coronary artery disease*, *CVD* Cardio-vascular disease, *DCM* Dilated cardiomyopathy, *HFrEF* Heart failure with preserved ejection fraction, *HT* Hypertension, *LVEF Left ventricular ejection fraction, LVH* Left ventricular hypertrophy, *MVR* Mitral Valve Regurgitation, *NSTEMI* Non-ST-Elevation Myocardial Infarction, *PAF* Paroxysmal atrial fibrillation, *PCOS* Polycystic ovary syndrome, *PAF* Paroxysmal atrial fibrillation, *PHT* Pulmonary hypertension

The following key themes were identified:

1. Access to a cardio-oncology clinic promotes information and understanding
2. The experience of early CVD intervention
3. Factors promoting integrated care
4. Balancing cancer treatment and CVD symptoms
5. Managing past and emerging CVD risk factors

### Access to a cardio-oncology clinic promotes information and understanding

Most participants reported they were unaware of the association between CVD and cancer treatment. Even at the time of diagnosis and treatment planning participant reflections suggested there were variances in understanding. More often women with breast cancer, who were being treated with Herceptin, reported they had been informed of CVD risk and this awareness was reinforced by regular ECG monitoring.

It [the risk of CVD] was [explained to me], but it was not emphasised. Yeah, I knew that there was some chance that I might develop some heart failure or some sort of cardiac irritation. (P9)

I was on the Herceptin and they knew that the Herceptin could mix with [affect] your heart. (P4)

Despite initial feelings of shock and anxiety when considering the risk of developing CVD alongside cancer, on reflection, many participants appreciated that cancer treatment could cause harm.

The logic inside my brain went, “Yeah, I guess that’s right”, you know. I guess it makes sense.… it [CVD] could happen. (P13)

I wasn’t aware that there was a likely or a possible side effect, but then there are so many other side effects, so it makes sense. (P15)

Most participants reported they were referred to the cardio oncology clinic at an early stage in their cancer treatment, due to the experience of existing CVD, or in response to emergent CVD symptoms. All participants expressed that early referral to the cardio oncology clinic provided a sense of relief and comfort rather than exemplifying any health concerns. There was a consensus across participants towards feelings of gratefulness and being “reassured” (P8) at close monitoring, such that if anything was detected, it could be treated early.

I sort of feel like a relief actually…. because I am being treated for cancer but I got these really good people looking after my heart as well. If something is going to happen there is a good chance that they can catch it - so that is a comforting thing really, more than confronting. (P50)

Participants were also grateful for close monitoring as their reports suggested they weren’t confident in their own ability to detect symptoms of CVD. As it was, several participants attributed emerging CVD symptoms to be side effects from chemotherapy.

But if I hadn’t been there [at the clinic], I don’t whether I would have noticed heart symptoms or when it would have been picked up or any of that sort of stuff. (P3)

But I never really noticed. I was not as active as what I usually was and I was tired, lethargic, I was sleeping a lot. I just generally felt quiet but I thought that I was feeling that way because of the chemo, not because of the heart. (P4)

### The experience of early CVD intervention

Most participants with a new diagnosis of CVD, reportedly felt confronted or “panicked” (P7) by the detection of CVD through altered blood results or imaging.

It was scary…. It is confronting, it is, and I know that heart disease takes a lot of people who have had chemo and cancer treatments. (P3)

Once enrolled in the cardio-oncology clinic, participants were notified of any CVD changes at an early stage. For example, one participant was notified of an elevatation in troponin by her General Practitioner (GP) and then promptly received a phone call from the cardio-oncology clinic to discuss treatment implications.

They did regular bloods and I got a phone call off my GP... I was like [shocked] but then I had a phone call from the cardio-oncology clinic and the cardiologist. (P11)

Following CVD changes all participants appreciated regular monitoring and ongoing management of CVD symptoms. Further, the responsiveness of the cardio oncology clinic and recommendations provided made them feel cared for. Being treated with a calming and reassuring manner helped to reduce any apprehension about their health.

It was great, well, [the cardiologist] is such a comforting and calming person. It was nice just to know that there was some support behind it, just to keep an eye on things and make sure I’m OK… and let me know what was happening. If there was a problem then we could start to deal with that as well. (P3)

The quality of clinicians’ explanations was perceived as a key factor in promoting confidence and trust in the treatment proposed.

I was quite worried, yeah. Didn’t really know what it [CVD changes] meant, but they were wonderful and [the cardiologist] explained it to me. (P7)

Quality of communication was closely linked with frequency of communication and available access to clinicians for information when needed.

I think just keeping me in regular contact and listening to what the expert is talking about. You have got to put your trust in them that they are doing the right thing. (P14)

I think they managed to check in with me with enough frequency to reassure me that my heart was doing okay. I have had some positive signs but knowing it [the clinic] was there was reassuring. But it was not invasive like I was not getting panicked because I was getting too many messages. So, everything sort of felt like they were just routine checks and that was reassuring. (P8)

This was particularly the case when participants, experiencing emerging or exacerbated CVD, received early intervention that promoted continuation of cancer treatment.

So, once my heart function gets below 45, my treatment has to stop and I have to wait for my heart function to go back up again. But [the cardiologist], has helped a lot, because he puts me on the medication to bring it up or whatever….. So, I can continue treatment. Happy days. (P6)

I developed Cardiomyopathy. I have 46% heart function at the moment, so it’s dropped. The chemotherapy has knocked it around a bit, but [the cardiologist] has got me on medication and monitoring me every 3 months or so. So far, everything is going okay. (P3)

### Factors promoting integrated care

All participants appreciated the collaboration and shared understanding they observed between their treating specialists, especially their oncologist and cardiologist. In response, participants felt that any specific questions they had could be better targeted by the specialists with the most expertise.

It has been really good because they all know what the other one is thinking and doing for me. I think it has been really encouraging. (P14)

I feel comfortable. [The cardiologist] has been an assurance that …. neither [specialist] was sort of guessing something out of their field. They are both excellent, you know. On occasions the oncologist might say in discussion, “Oh that’s [the cardiologist problem]” or [the cardiologist] will say, “Go talk to [the oncologist about that].”…. I talk to both of them. (P13)

Indeed, one participant expressed that knowing their specialists were in collaboration meant they didn’t have to keep repeating things.

Yeah, great, they know things before I even have to say something. They are on top of it, which is really good. It makes me feel good because I don’t have to sort of keep repeating myself over and over. (P6)

Participants also reported that collaboration between specialists made them feel like they were being treated as people rather than patients, since they felt they were an integral component of treatment decision making.

They sort of did not make me feel like they were taking care of me as a pathetic little piece of meat, that needed to be turned back into a human. They sort of gave me this feeling that they were part of the journey, they were sort of coming along and supporting me. It sounds pretty cliché but it seemed really real to me. (P8)

I was really happy that [the cardiologist] gave me that option [of starting medication or not]. I felt really empowered. It wasn’t just like, you are a patient and I am saying this is what we should do… he did assure me that even though there is a marker of damage, he has seen a lot higher and he still gave me the option. (P11)

Overall, participants felt comforted that everything that could be done was being done.

It is comforting to know that we have doctors and specialists that do not give up you know. They are not just going to say, “Sorry you are not a candidate anymore for treatment” - they can manage it for as long as they kind of want to, so it is kind of comforting. (P11)

### Balancing cancer treatment and CVD symptoms

Participants readily expressed the challenges of dealing with the treatment demands of two chronic diseases, namely cancer and CVD. Many reported that discerning which disease took precedence was a daily battle depending on their symptom presentation.

Having two [disease] can be daunting ... I mean the last thing you think of before you go to sleep is like, “Let’s hope I wake up tomorrow”. Or, “I wonder how the cancer is going to be tomorrow?” or you know, “How will my heart be?”. (P1)

I can’t isolate them [what disease is most pressing] any way. I just try and look after each of those conditions, yeah. (P13)

Many participants indicated that responding to treatment and was a balancing act that depended on presenting symptoms and which treatments could be maintained. For most part, overcoming cancer was the priority.

I don’t know, it’s really hard to find that balance, because if my heart function drops then I miss out on treatment…. it is sort of scary to not have your treatment. It’s just that, it is a double-edged sword, because my cancer is sort of stable, but my heart isn’t, so, you just can’t win. It would be good if it all just came together and you know, if it is not one thing it’s another, but yeah, finding a balance… I haven’t found it yet. It’s really really hard. But they keep you stable, that is fantastic. (P6)

It’s just a balancing act …. I honestly couldn’t tell you who would take precedence. (P3)

Many participants reported that balancing the medical demands of two conditions was overwhelming, especially due to the frequency of appointments.

I can show you my diary, it’s just full and I have to keep this diary and I write stuff up on the white board as well. It just blows my mind the amount of appointments I have got. So, I often feel a bit overwhelmed. (P7)

I need to book things in, juggle everything – brain scan, PET scans. It is hard dealing with everything at once. (P2)

Most participants expressed comfort and relief in knowing that treatment of the CVD meant their heart was stabilised so that they could continue active cancer treatment.

Oh, absolutely it has been hard. As I said [cardiologist] is doing a great job with my heart. So, thank God that is not a problem and it is not constantly a problem. Because he has done a great job, it is giving me time to take my mind off the heart. I know it is still there and it will never go. The oncologist currently drives everything because the heart has been sort of stable and has not been changing. (P5)

But I don’t really feel anything [CVD symptoms], the tablets seem to be doing the job [the cardiologist]. I am really pleased … because then I can concentrate on the cancer. I think it is more important being positive with the cancer than your heart. The heart is okay, I know it is important but it’s not hurting as much as what the cancer is so. (P1)

### Managing past and emerging CVD risk factors

Participants reflected on risk factors that were diagnosed both before and after their cancer diagnosis.

I was stressed all the time and they told me that stress would have had a lot to do with my cancer. (P1)

I am aware of my weight gain and the normal lifestyle ones I suppose that anyone gets at my age. But I have had no high blood pressure or anything like that. (P3)

Making an active effort to address CVD risk factors following cancer diagnosis was experienced on a spectrum whereby some participants made deliberate attempts and others were less likely to.

Yeah, I have cut back alcohol. I was a moderate drinker – I probably had a bottle of wine on Friday, Saturday and Sunday and now I do not. I still have drink but I do not drink during the week. I am eating really clean and healthy that is a big thing for me. (P4)

For many participants the side effects of their cancer treatment, especially fatigue, left them with little energy to address risk factors.

I do not exercise enough, I know that, but when you are exhausted all the time. It is hard to do that, yeah. I cannot say that I prioritised one over the other. It was a bit of a blow to get the extra diagnosis, but it was what it was, there was nothing you could do about it. Just suck it in and soldier on, you know. (P15)

I feel really fatigued, I am still coughing a bit. I actually do not think I am at the level where I feel that I can start recovering. I am still way below that level yet, but my sense of humour is starting to return. (P8)

Previously active participants expressed feelings of frustration when cancer side effects got in the way. In general, they tried to keep active with valued activities such as walking and house renovations.

I used to be on the go, sort of bushwalking and go upstairs and things like that without puffing. This is ridiculous… I will just try to my breath again and just continue. I know my friends and family wouldn’t care, they would hang back and wait for me. It bothers me more than anyone else. (P6)

I feel in myself that I am slowing up, that’s quite noticeable with the amount of work I can do, yeah. I suppose that’s normal, with not doing as much, I can feel my strength weakening. When I do go to do something, it takes longer or I need assistance to do it. (P13)

Following their cancer diagnosis, participants reported varying experiences about receiving education towards managing CVD risk factors. A few participants reported the value of participation in an Information Day for newly diagnosed cancer patients prior to commencing chemotherapy.

I went down for an information day so that [managing risk factors] was highlighted then. (P4)

However, some patients fell through the gap towards with receiving details of the Information Day, such as during admission to hospital.

Before you start chemo, they give you an information session the day before. Well because I was an inpatient, I did not get that. (P15)

Overall, the majority of participants reported they had received little or no education towards risk modification of CVD symptoms by their treating clinicians, except for one participant who was a smoker. However, despite being told not to smoke she reported she “wasn’t referred for professional support.” (P15)

No-one has really talked to me about risk factor modification. (P6)

One of the doctors said early on how important it is to stay active. She said [its important] to keep moving because the chemo goes in, it does what it needs to do and then you have to make it leave the body….. cardio or weight lifting, just gentle exercise, moving, gentle swimming if you can do that. I think it is very important to keep doing that, I walk the dogs most days. (P11)

Several participants were encouraged to exercise by their colleagues and friends, mostly as a way to promote better cancer outcomes. For these participants the accountability of friends was reported to be strong motivator for maintaining exercise during and after treatment.

The best bit of advice that was given to me was by one of my colleagues, a physiotherapist, who said, “The research suggests that physical activity contributes to good cancer outcomes.” So, I continued to walk a lot and get out of the house, and every day my mantra was get out, get dressed, put your lipstick on, get out of the house, and rain, hail or shine, maybe not hail, I would walk. Depending on what’s happening with my shortness of breath and ankle oedema, I had to cut out the hills, but I always walked. (P9)

I ran into one of the girls I used to go to the gym with. She knew what was going on and she said, “When are you coming back, come on come back”. Then the trainer texted me and said, “Do you want to come back and are you ready for this?” I said, “Yes.” I haven’t missed a day. (P4)

Weight management concerns were commonly-expressed by participants as a mechanism to reduce their CVD, however some participants indicated they were focusing on recovery and would address CVD down the track.

I am 100 k, I already know that that puts pressure on my body and the treatment from cancer has limited my physical activity. I just need to work through one thing at a time and just work on that to better myself. (P10)

For others the diagnosis of cancer provided a reason to reflect on lifestyle and make changes.

Yeah and when I used to work, I’d work late at night and so sometimes I didn’t come home till 11 at night. I hadn’t had diner so I’d stop by and I pick up Maccas (McDonalds) or I’d come home and have vegemite on toast with lots of butter. I was just in that rut. That is why now with my work I don’t want to go out and do any of that anymore. This is my time to get myself back. So, I am eating really well, exercising. (P4)

Cancer afforded me the opportunity to get off the treadmill and step away from doing somethings. Perhaps it was time to… re-evaluate… (P9)

## Discussion

This study explored patient perceptions toward acceptability and feasibility of a Cardio-oncology services and its impact on integrated care. Results highlighted the benefit of rapid access (usually within 1 week for outpatient referrals (see Introduction) and on the day of referral for inpatient referrals) to a cardiologist (co-located with patient’s usual cancer services) for management of existing or emerging CVD symptoms in the context of cancer treatment. Participants valued the opportunity for regular monitoring and management of CVD issues that enabled them to continue cancer treatment. Results are of clinical importance since comorbidity, such as CVD, can affect timing of diagnosis, timing, choice and duration of cancer treatment, and outcomes of people with cancer [24]. Overall, given the paucity of research exploring how people perceive Cardio-oncology, this study sheds light on how people would like to have Cardio-oncology services delivered and provides insights into how services can better align with models of integrated care. Despite the well documented importance of risk factor management for chronic disease, we identified gaps in integrated cared including the provision of education and support towards making positive lifestyle changes that reduce the risk of CVD in cancer patients.

Key results from this study identified that early access to a cardio oncology services improved participant understanding of the association between cancer treatment and cardio-toxicity. Early access to CVD treatment also assisted in reduction of cardiovascular complications, improve recovery of cardiac function, leading to better cardiovascular outcomes, while at the same time ensuring the continuation of appropriate cancer treatment, leading to better cancer-related outcomes, as has also been demonstrated in other evaluations of cardio-oncology services [25]. This is an important finding since people are now living with or surviving cancer due to continuous progress in cancer management. In response, current health service delivery is shifting from a single disease treatment paradigm to a holistic paradigm, whereby cancer treatment is no longer provided without consideration for the full context of the person [26]. This has been a long and ongoing process with past research also showing that patients experiencing cancer and other comorbidities experienced fragmentation of care and unmet needs [27, 28].

Participants in our study reported that access to a cardio-oncology service promoted coordinated, integrated care and feelings of personalised care. This was underpinned by feeling valued as a person, such as being given choice in decision making and not having to having repeat their experience to multiple clinicians, factors consistent with the concept of integrated care [29]. While there is no widely accepted definition of integrated care, it is commonly referred to as an approach to overcome health care fragmentation with the aim of achieving ‘improved patient care through better coordination of services’ [30]. A central feature of integrated care is communication whereby skilled information exchange promotes effective patient self-management [31], helping patient feel valued and that their preferences are heard. A pleasing finding of this research was that patients felt their needs were included in multidisciplinary discussions and in response this promoted feelings of confidence in treating professionals and the care they received.

Most participants in this study already had baseline risk factors for CVD and with evidence that CVD is more prevalent in individuals with cancer, many patients would fall into this category [32]. Additional concern stems from the fact that patients with cancer who develop CVD experience worse outcomes [33]. While the establishment of a cardio-oncology service allows for CVD risk assessment we identified gaps in facilitating participants to address CVD risk-reducing behaviour (weight management, diet, alcohol, engaging in physical activity). While some participants perceived the emergence of CVD as a reason to address risk factors, common to cancer and CVD, the majority struggled to make changes, especially during cancer treatment. This is not surprising and previous research has identified that patients with comorbidities find it difficult to prioritise health outcomes but place valued on being independent and staying alive [33, 34].

There is growing interest in how to deliver CVD risk management programs that address risk reduction strategies. Targeted CVD programs help patients make lifestyle changes, monitor symptoms, and promote treatment adherence in order to prevent disease progression and reduce health complications. In addition, risk management programs facilitate patient empowerment and activation whereby people have the capacity and confidence to manage their health and health care. However, most CVD programs have been conducted during the survivorship phase of cancer care and there has been less focus on what is helpful during the active treatment phase [24]. To date, self-management programs such Chronic Disease Management Programs (CDMPs) have been found to be helpful among those with chronic conditions [35–37]. Growing research demonstrate the accept acceptability able and feasibility of self-management programs for cancer survivors [38, 39]. However, these are not widely made available and there is scope to consider how they can better align with cardio-oncology services. As such, future research should explore mechanisms that promote management of CVD risk factors in people with cancer such and education and programs that help promote lifestyle change during and after treatment.

## Conclusion

Qualitative interviews suggest that early access to a cardio-oncology service promotes knowledge, while facilitating early identification of changes in cardiac state or emerging CVD. However, our findings suggest that more can be done to promote integrated of care that addresses CVD risk factors. Early intervention was shown to mitigate CVD and associated complications while also ensuring the continuity of cancer treatment, more support is needed to address CVD risk factors such as a access to a Chronic Disease Management Programs.

## Data Availability

Available on request.

## Abbreviations

CV: Cardiovascular
CVD: Cardiovascular Disease
LVEF: Left ventricular ejection fraction
P: Participant
